# Unusual Late Relapse of ALK-Positive Anaplastic Large Cell Lymphoma Successfully Cleared Using the ALK-Inhibitor Crizotinib: Case Report

**DOI:** 10.3389/fonc.2020.585830

**Published:** 2020-10-02

**Authors:** Dennis Christoph Harrer, Karin Menhart, Stephanie Mayer, Wolfgang Herr, Albrecht Reichle, Martin Vogelhuber

**Affiliations:** ^1^Department of Medicine III – Hematology and Internal Oncology, University Hospital Regensburg, Regensburg, Germany; ^2^Department of Nuclear Medicine, University Hospital Regensburg, Regensburg, Germany

**Keywords:** crizotinib, anaplastic large cell lymphoma, ALK, relapse, hematology

## Abstract

Anaplastic large cell lymphoma (ALCL) with ALK-translocation constitutes an aggressive lymphoma with high sensitivity to anthracycline-based chemotherapy. Relapse, however, is observed in about one-third of patients. Salvage treatment incorporates high-dose chemotherapy followed by autologous or allogeneic stem cell transplantation, treatment with the CD30-specific immunoconjugate Brentuximab vedotin (BV) and the use of ALK-inhibitors, such as crizotinib. In this case report, we present a patient with a rare late relapse of ALK-positive ALCL following chemotherapy, who was neither eligible for high-dose chemotherapy nor treatment with BV. Relapse therapy was carried out with daily crizotinib, which rapidly mediated complete regression of all ALCL manifestations. In light of few clinical trials published on the use of crizotinib against ALCL, we want to further substantiate the efficacy of crizotinib as salvage therapy in patients with relapsed ALCL especially if ineligible for high-dose chemotherapy or BV treatment. Finally, we would like to enhance vigilance for potential late relapse of ALCL more than a decade after frontline treatment.

## Introduction

Anaplastic large cell lymphoma (ALCL) is a rare hematological neoplasia representing only two percent of all non-Hodgkin’s lymphomas (1). Approximately 50% percent of patients with ALCLs bear a translocation of chromosome two and five t(2;5) fusing the N-terminal part of the protein nucleophosmin (NPM) to the kinase domain of anaplastic lymphoma kinase (ALK), which results in permanent tyrosine kinase activity (1). ALK-positivity is not unique to ALCL but can be found in various other tumor entities, e.g., in subgroups of non-small cell lung cancer (NSCLC) (2).

ALK-positive ALCLs are usually aggressive T cell lymphomas with high sensitivity to chemotherapy. Hence, first-line treatment of ALK-positive ALCLs is predicated on anthracycline-based multi-agent cytotoxic protocols, such as CHOP, CHOEP and A + CHP (1, 3). Nevertheless, relapse, usually occurring within the first 5 years after first-line chemotherapy, is observed in about one-third of patients (1). Currently, salvage strategies for relapsing or refractory ALK-positive ALCLs rely on platinum-based regimens, high-dose chemotherapy followed by autologous or allogeneic stem cell transplantation and the CD30-specific immunoconjugate Brentuximab vedotin (BV) (1, 4). Additionally, targeted therapy using ALK inhibitors, such as crizotinib, has attracted increasing attention over recent years.

Crizotinib is an ATP-competitive multi-tyrosine kinase inhibitor (blockade of ALK1, ROS1, and MET1) (5). Currently, FDA-approval is still restricted to subgroups of NSCLC. Regarding ALCL patients the safety and clinical efficacy of crizotinib was initially showcased in an early-phase pediatric clinical trial (6). In adults several clinical courses of successful ALCL regression upon use of crizotinib at various disease stages have been published (7–11). Importantly, given the paucity of clinical trials evaluating the efficacy of crizotinib in adult ALCL patients, clinical evidence and practical advice regarding the use of crizotinib in adult ALCL patients is still significantly sourced from a steadily growing number of case reports.

In this case report, we present a patient with a rare late relapse of ALK-positive ALCL following anthracycline-based chemotherapy, who entered ongoing complete remission after initiation of treatment with crizotinib. In addition, this report aims to further corroborate the clinical efficacy of crizotinib in the treatment of relapsed or refractory ALK-positive ALCL. Finally, we would like to highlight the possibility of unusual late ALCL relapse occurring more than a decade after successful first-line therapy.

## Case Presentation

**FIGURE 1:**
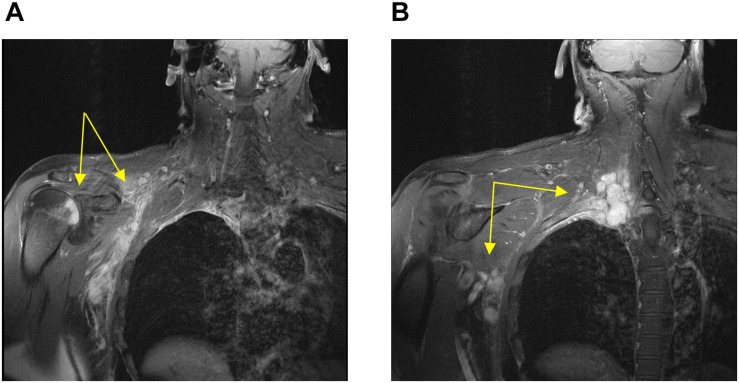
Magnetic resonance imaging conducted at first diagnosis showing soft tissue-masses (yellow arrows) with infiltration of the right humerus **(A)** and the right pleura **(B)**.

**FIGURE 2:**
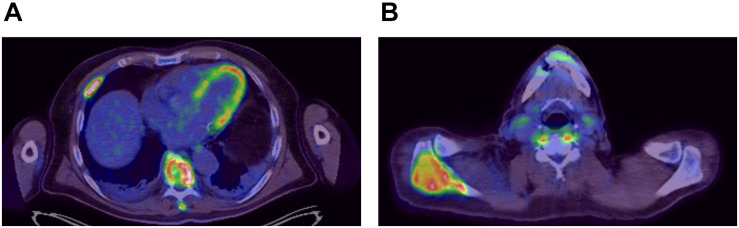
FDG-Positron emission imaging at first relapse of ALCL showing hypermetabolic lesions around the spine **(A)** and within the right scapula **(B)**.

**FIGURE 3:**
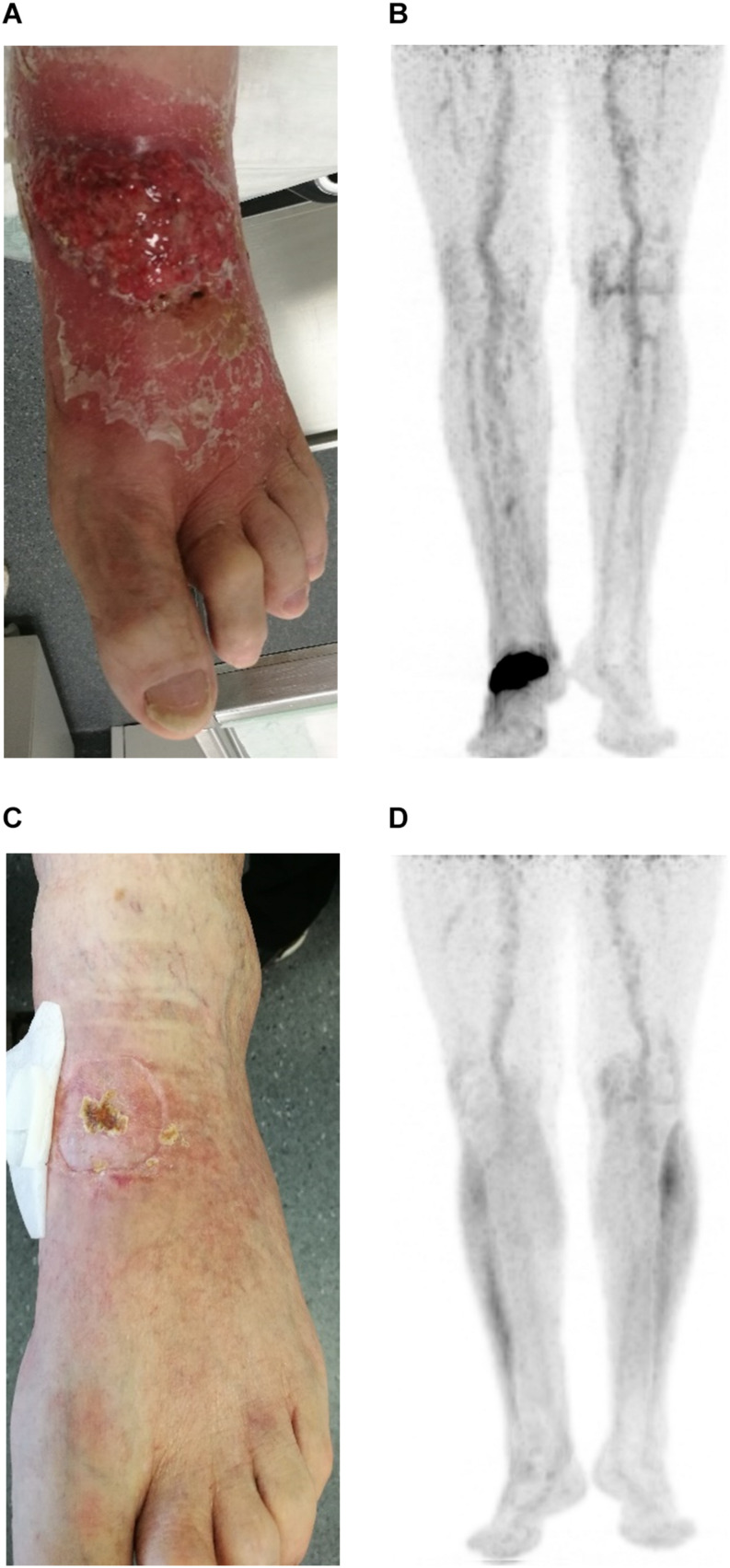
Photographic documentation and positron emission imaging at second relapse of ALCL **(A,B)** and at 3 months after start of crizotinib treatment **(C,D)**.

A 54-year-old previously healthy man complained about pain in the right upper back. Physical examination revealed a palpable induration located at the right shoulder. Magnetic resonance imaging (MRI) showed a tumor mass with infiltration of the right humerus (Figure 1A) and the right pleura (Figure 1B). After computed tomography (CT)-guided biopsy and subsequent histological examination, the diagnosis of anaplastic large cell T cell lymphoma (ALCL) was made. The ensuing molecular analysis revealed the presence of a translocation t(2;5) indicating ALK-positive ALCL. Furthermore, bone marrow biopsy showed infiltration with lymphoma cells. Therapeutically, chemotherapy according to the CHOEP-14 protocol (cyclophosphamide, doxorubicin, vincristine, etoposide, prednisone administered biweekly) was started. After eight cycles of chemotherapy, the patient entered into a complete remission without residual ALCL presence visible on CT-imaging and bone marrow examination. During chemotherapy, no relevant side-effects, such as serious infections or polyneuropathy, were observed. Following 14 years of normal follow-up care without any signs of relapse, the patient reported nighttime sweating accompanied by fever and increasing pain emanating from the left thorax. FDG-Positron emission tomography (FDG-PET) revealed multiple hypermetabolic osteolytic lesions affecting the spine (Figure 2A), several ribs (Figure 2A) and the right scapula (Figure 2B). Additionally, pathological tracer uptake was observed in periaortic and iliac lymph nodes. CT-guided biopsy of a hypermetabolic lesion located in the eighth rib of the left hemithorax revealed a relapse of the previously known ALK-positive ALCL nearly 14 years after chemotherapy. Due to the long time to relapse chemotherapy with CHOEP was repeated after a brief cyclophosphamide pre-phase. Moreover, denosumab was added for skeletal support. Upon six cycles of chemotherapy FDG-PET-imaging showed complete regression of all ALCL lesions. Fifteen months later, the patient presented to the emergency room with fever and pain in the left foot. Physical examination revealed a red and swollen left forefoot with tenderness upon palpation (Figure 3A). Magnetic resonance imaging of the left foot showed a cutaneous mass without involvement of muscles or bones. Dermatohistopathological examination of skin biopsy confirmed the second relapse of ALK-positive ALCL. Subsequent staging via FDG-PET revealed tracer uptake in the left forefoot (Figure 3B) as well as pathological lymph-nodes in the left groin. No serious comorbidities were present apart from vincristine-induced sensory polyneuropathy (paresthesia and digital numbness without serious limitations in instrumental activities of daily living corresponding to polyneuropathy grade 2), which started after the last cycle of relapse chemotherapy. At second relapse, the patient was not considered eligible for high-dose chemotherapy due to advanced age coupled with significant cumulative toxicity from previous therapies. After comprehensive discussion of the possible options for salvage therapy, the patient declined BV-treatment due to a pronounced fear of an exacerbation of pre-existent grade two polyneuropathy arising most likely from cumulative vincristine toxicity. Hence, off-label treatment with the ALK-inhibitor crizotinib was initiated at a dose of 250 mg twice a day. Over the course of 3 months the cutaneous lesion on the left forefoot vanished (Figure 3C), and FDG-PET-staging showed metabolic complete remission (Figure 3D). So far, the patient’s complete remission has been maintained for 9 months and is still ongoing at the time of this writing. Crizotinib treatment will be continued until disease progression.

## Discussion and Conclusion

Anaplastic large cell lymphoma (ALCL) with ALK-translocation is a rare subgroup of non-Hodgkin’s lymphoma with favorable response to chemotherapy (1). Nevertheless, in case of relapse, survival rates drastically decrease (1). For patients suffering from relapsed ALCL high-dose chemotherapy followed by autologous or allogeneic stem cell transplantation confers a 2–5-year progression free survival rate of 30–60% (12). However, due to comorbidities and age not all patients are eligible for stem cell transplantation. Another salvage therapy for relapsed ALCL is represented by the CD30-targeting immunoconjugate Brentuximab vedotin (BV). In a multi-center phase II clinical trial, the use of BV achieved an overall response rate of 86% and a complete remission rate of 57% in patients with relapsed or refractory systemic ALCL irrespective of ALK-translocation status (4). In total, polyneuropathy with sensory disorders was observed in more than 50% of BV-treated patients. However, the rate of severe polyneuropathy (grade 3 and 4) was only 9–12% after BV-treatment (3, 4). Owing to the peculiarly late relapse the patient in this case report was first re-exposed to frontline chemotherapy, which initially mediated rapid regression of all ALCL lesions. The decision to repeat chemotherapy instead of switching to BV-treatment, which represents the standard treatment for relapsing ALCL, was mainly based on the outcome of the first chemotherapy treatment, which rapidly mediated long-lasting complete remission without causing any serious complications. At second relapse, the patient was highly worried about a possible exacerbation of his pre-existent grade two polyneuropathy by BV-treatment. Albeit the risk of severe polyneuropathy with tangible repercussions on daily activities is only observed in approximately 10% of patients treated with BV, the patient opted for off-label use of the ALK-inhibitor crizotinib. Dose-intensive therapies, such as autologous or allogeneic transplantation, were discarded owing to advanced age and cumulative toxicities from previous treatments. While the efficacy of ALK-inhibitors in NSCLC was confirmed in several clinical trials resulting in FDA approval for crizotinib for the treatment of ALK-translocated NSCLC, studies on the efficacy of ALK-inhibition in ALCL are still scant, and largely obtained from case reports and some early-stage clinical trials. The first data highlighting the clinical efficacy of crizotinib in ACLC were shared in 2011 demonstrating swift complete remissions following treatment with crizotinib in two heavily pre-treated patients with ALCL (8). Another report on eleven patients with chemorefractory ALK-positive ALCL treated with crizotinib as monotherapy showed an overall response rate of 90.9%, a 2-year overall survival rate of 72.7% and a 2-year progression free survival rate of 63.7% (7). In two pediatric clinical trials, crizotinib was found to achieve rapid complete remission of ALCL lesions in more than half of all treated patients (6, 13). A phase 1 study evaluating crizotinib in adult ALCL patients showed an overall response rate of 52.9% with eight complete responders and one partial responder (9).

Recently, activation of the IGF-1R pathway was described as an important mechanism conferring resistance to crizotinib activity on ALCL cells, which could be counteracted by the concomitant use of IGF-1R pathway inhibitors (14). Moreover, the mutation NPM-ALK G1269A was identified as another cause underlying crizotinib-resistance of ALCL cells. Importantly, structurally different second-generation ALK-inhibitors, such as ceritinib, could inhibit the growth of NPM-ALK G1269A mutated cells and could serve as an important salvage therapy for ALCL with crizotinib-resistance (14). Clinical efficacy of ceritinib, especially the potential to induce long lasting complete remissions, in patients with ALK-positive ALCL was initially demonstrated by Richley et al. (15).

Against the backdrop of this still maturing landscape of evidence further case reports and clinical trials are required to further substantiate the broad application of crizotinib in patients with relapsed or refractory ALK-positive ALCL. The patient in this case report is intended to receive crizotinib continuously until disease progression. Given the possibility of abrupt relapse of ALK-positive ALCL following discontinuation of crizotinib (16), continuous exposure to the ALK-inhibitor may be advantageous.

In conclusion, we report rapid complete regression of nodal and extranodal manifestations in a patient with ALCL in response to crizotinib treatment. In light of few clinical trials published on the use of crizotinib against ALCL, we want to highlight the efficacy of crizotinib as salvage therapy in patients with relapsed ALCL not eligible for high-dose chemotherapy or BV treatment. Finally, we would like to enhance vigilance for potential late relapse of ALCL more than a decade after frontline treatment.

## Data Availability Statement

The original contributions presented in the study are included in the article/supplementary material, further inquiries can be directed to the corresponding author.

## Ethics Statement

Written informed consent was obtained from the individual(s) for the publication of any potentially identifiable images or data included in this article.

## Author Contributions

MV and AR guided the treatment of this case. DH drafted the manuscript. KM provided the positron emission imaging material. WH and SM interpreted data and critically revised the manuscript. All authors read and approved the final manuscript.

## Conflict of Interest

The authors declare that the research was conducted in the absence of any commercial or financial relationships that could be construed as a potential conflict of interest.
